# Multilocular Lipoma in the Middle and Index Fingers: A Case Report

**DOI:** 10.7759/cureus.22172

**Published:** 2022-02-13

**Authors:** Tuqa A Alsinan, Tareg M Alhablany, Hesham R Alokaili, Abdulla S Altamimi, Mohammed Ehsan Rashidi

**Affiliations:** 1 Department of Plastic Surgery and Burn Unit, King Saud Medical City, Riyadh, SAU

**Keywords:** hand surgery, lipoma of middle digit, lipoma of index digit, hand lipoma, multilocular lipoma

## Abstract

Lipomas are the most common type of soft tissue tumor, and 95% of them are benign. While lipomas can present anywhere on the body, 1% of them are found in the fingers. The ultimate goal of management is surgical excision of the mass with preservation of the neurovascular surroundings. Here, we present the case of a 24-year-old, morbidly obese Saudi female patient complaining of large non-tender lumps in the index and middle fingers involving the palmar and dorsal surfaces of the left non-dominant hand. The lumps were associated with paresthesia and tingling sensations. The article aims to report and highlight the satisfactory outcomes after total excision of such lipomas and restoring the function as well as the cosmetic results of the hand.

## Introduction

Lipomas are benign fat cell tumors that are primarily found in the head and neck, as well as the shoulder and back [1,2]. Lipomas are considered to be the most common type of soft tissue tumor, and 95% of them are benign [1,3]. While lipomas can present anywhere on the body, 1% of them are found in the fingers [3]. Lipoma of the hand and fingers is considered to be rare; it usually affects the hypothenar and thenar regions [2]. The clinical presentation varies depending on the exact location, but it usually presents as a painless and soft mass felt under the skin [3]. In most cases, lipomas are not treated; they are only observed clinically unless the patient is symptomatic or aiming for cosmetic results [3]. The ultimate goal of management is surgical excision of the mass with preservation of the neurovascular surroundings [1-3].

## Case presentation

A 24-year-old, morbidly obese Saudi female patient presented with large non-tender lumps in the index and middle fingers involving the palmar and dorsal surfaces of the left non-dominant hand. The patient reported a history of the initial appearance of the lumps at the age of eight and surgical excision four years before presentation, as well as the history of debulking procedure three years before the surgical excision. The lumps were asymptomatic until they started to be associated with paresthesia and tingling sensations of the involved digits, which affected her daily activities. She does not smoke and is not exposed to second-hand smoke. The rest of her history was unremarkable. The physical examination results were all within the normal limits, except she had a limited range of motion due to the mass in the affected digits. Soft-tissue hemangiomas, vascular malformation lipoma, and lipomas were all considered as differential diagnoses based on the history given.

**Figure 1:**
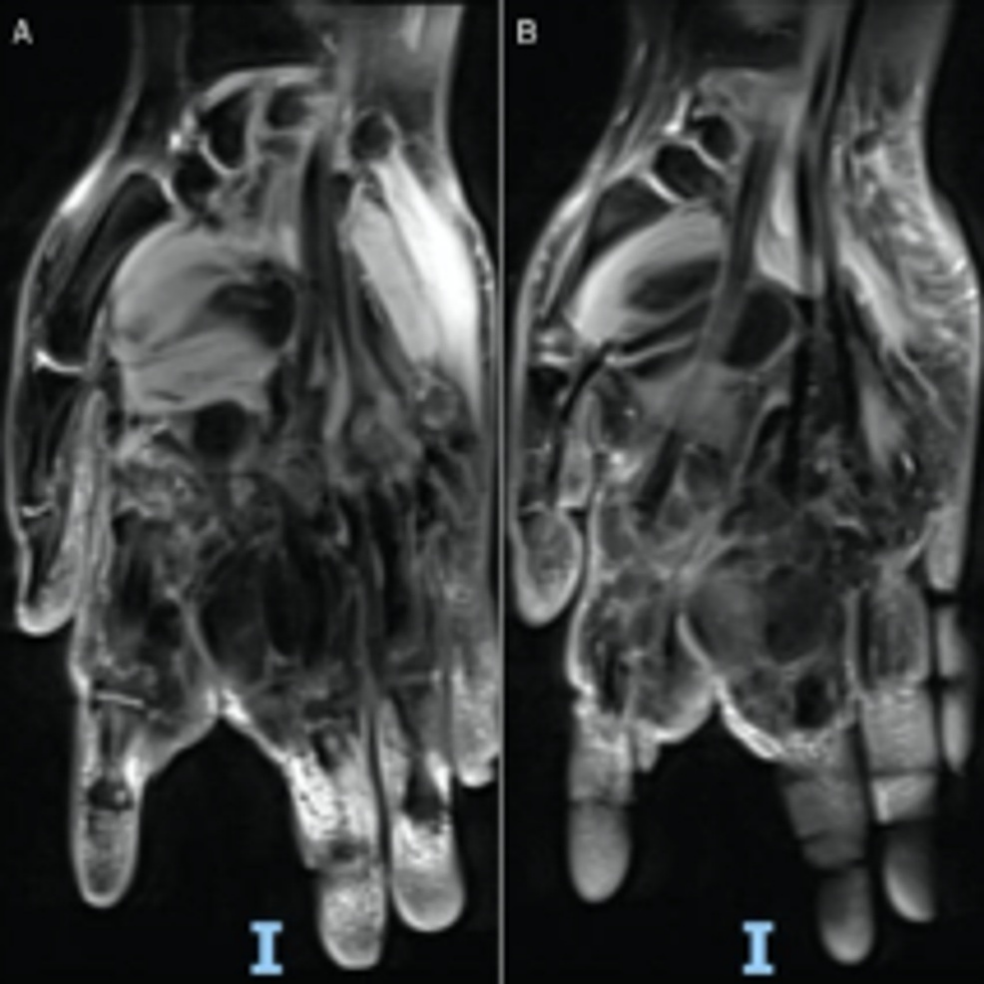
A and B are MRI-T1 images of the left hand showing the lipoma of the index and middle digits.

For further investigations, she underwent Magnetic Resonance Imaging (MRI), which showed an encapsulated multilocular mass in the proximal and distal sections of the index and the middle digits, extending to the palmar and dorsal aspects of the left hand until the carpal bone dorsally and from the first web space to the fourth web space with a volar extension of approximately 3 cm to proximal palmar crease [Figures 1A, 1B].

**Figure 2:**
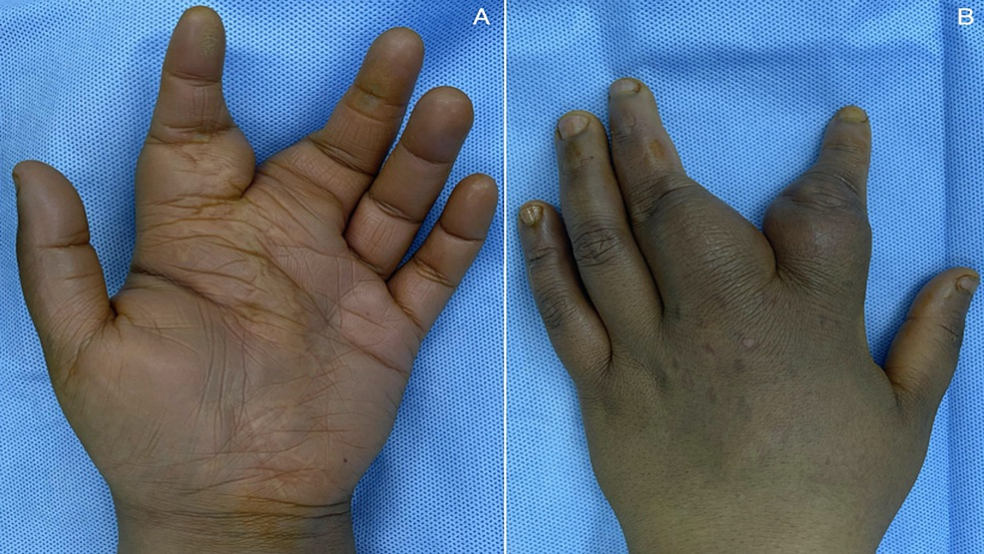
Preoperative photograph of the lipoma over the volar aspect of the left hand in image A. Dorsal view of the hand in image B.

The patient was booked and taken to the operating theatre under general anesthesia and full aseptic technique. Soft, mobile, raised, and fluctuant masses were felt over the second and third digits of the left hand with no overlying skin changes [Figures 2A, 2B].

**Figure 3:**
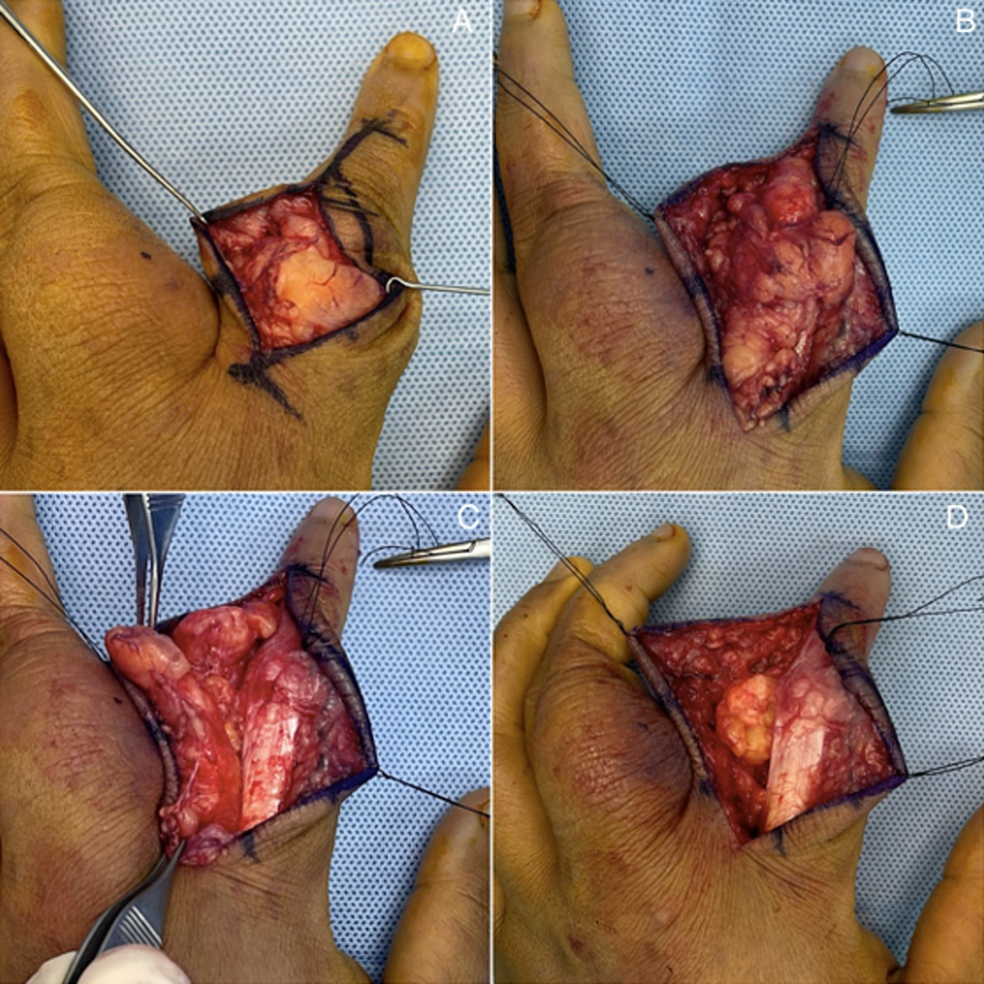
(3A, 3B, 3C, 3D). Segmental resection of the lipoma in the index digit. Incision of the dorsal PIP creases distally to the second web space’s dorsal center proximally.

Two lipomatous tumors were felt within the ulnar border of the index finger and the radial border of the middle finger. The incision spanned from the superior border of the dorsal proximal interphalangeal (PIP) creases distally to the second web space’s dorsal center proximal to the index digit. The incision was kept dorsal to the glabrous line [Figures 3A-3D].

**Figure 4:**
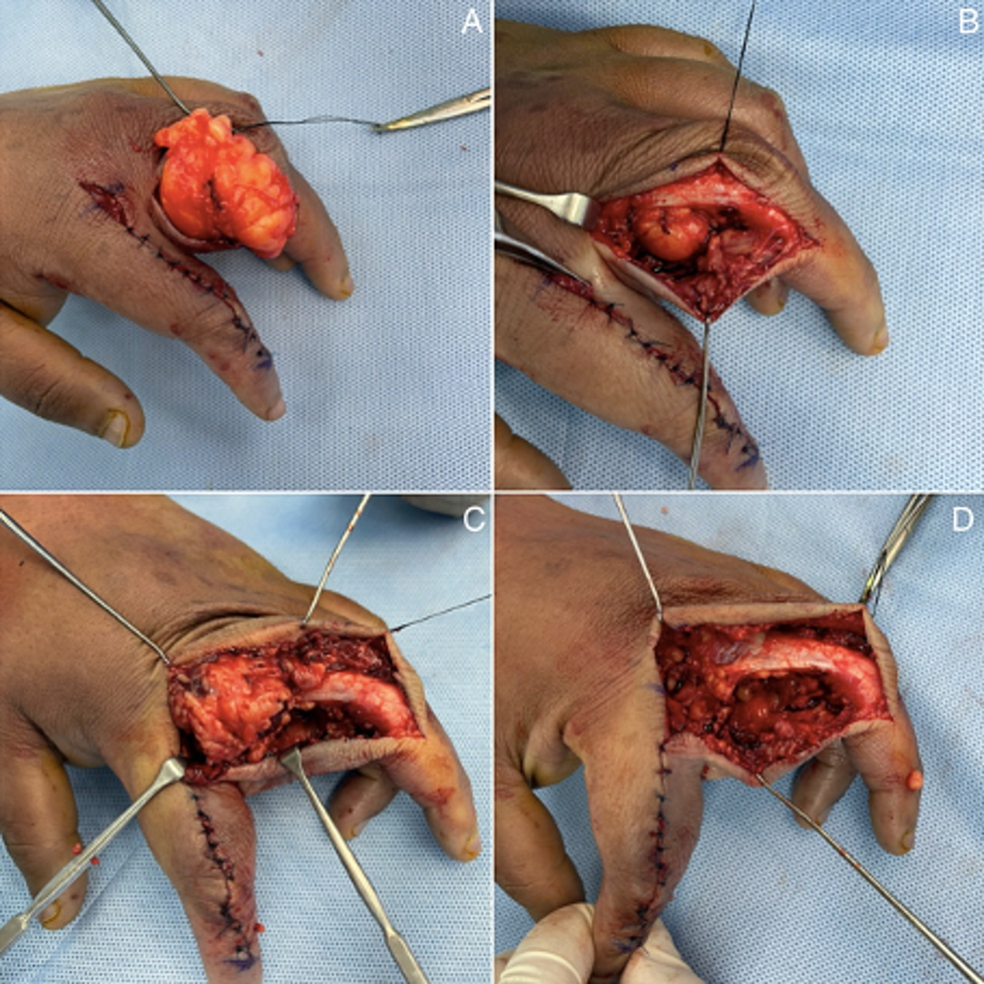
(4A, 4B, 4C, 4D). Segmental resection of the lipoma in the middle digit.

The lipoma was found to encompass the zone of incision and was adherent to the extensor mechanism; it was also engulfing the neurovascular bundle. Thus, segmental excision rather than en-bloc resection was preferred due to the engulfment of the structures. Similarly, another incision was made over the middle digit in the radial border, and extensor tendons were repaired, respectively [Figures 4A-4D].

**Figure 5:**
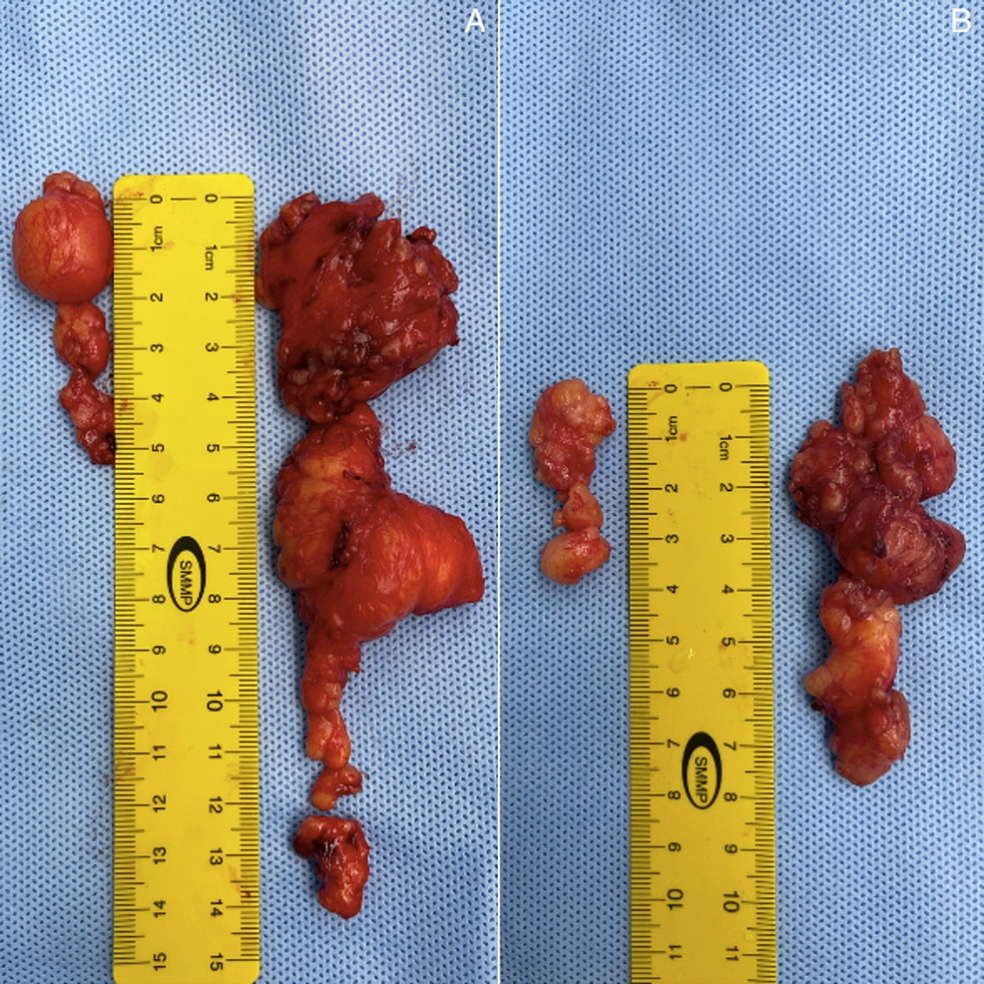
Lipoma excised from the index digit in image A. Lipoma excised from the middle digit in image B.

The tumor on the middle digit had similar proportions, distribution, and attachments as the tumor on the index digit [Figures 5A, 5B]. 

**Figure 6:**
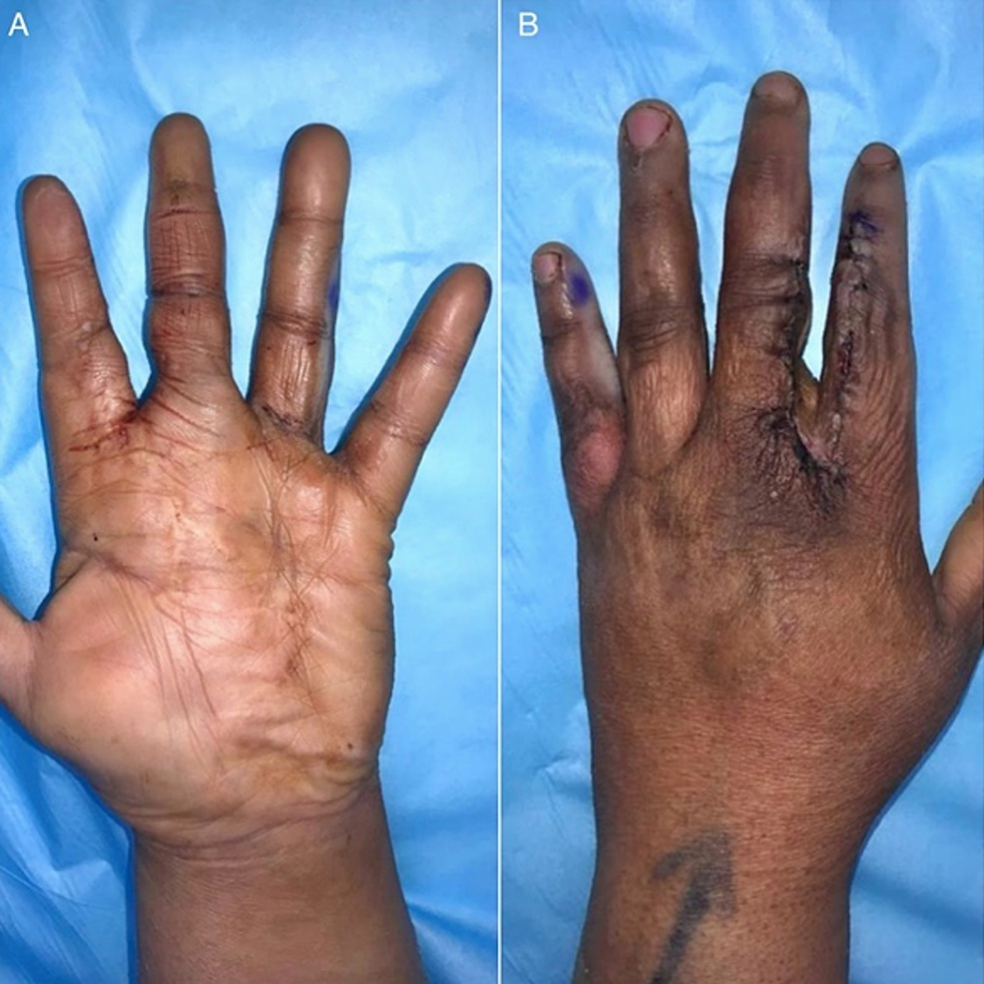
One week follow-up post operative. Volar aspect in image A. Dorsal aspect in image B.

Intra-operative findings were summarized as two lipomatous tumors excised from the index and middle digits over the ulnar and radial borders, respectively. Histopathology revealed that the specimens consisted of several fragments of fibro-fatty tissue measuring 7.5 x 5.5 x 1 cm, and three ovoid pieces of yellow-tan tissue measuring 5.5 x 4 x 3 cm, and 2.5 x 2 x 1 cm, respectively, for both the left index and left middle digits. After the procedure, the patient was placed in a compressive dressing and volar splint. The patient tolerated the procedure very well and reported satisfactory outcomes with no complications at the one-week follow-up appointment at an outpatient clinic [Figures 6A, 6B].

## Discussion

Lipomas are the most common form of benign soft tissue tumors with a rare incidence rate in the fingers [1-3]. They can be observed or treated with surgical excision, depending on the patient’s symptoms and the cosmetic goal [3]. Several articles in the literature have reported on the rarity of lipomas in the hand and digits. Recently, two cases were published reporting on a multilocular lipoma of the left thumb and a giant lipoma of the index extending to the dorsum of the right hand that was excised by performing a modified Bruner incision technique [1-3]. The two cases resulted in a successful surgical outcome with good cosmetic results [1-3]. Lipomas in the middle finger are rare, and excision is the best way to restore the normal function of the digit. Considering lipoma of the finger as a differential diagnosis and the preoperative use of ultrasound and MRI aid in good surgical outcomes, which is highlighted in the literature [2-5]. Furthermore, a rare case of a giant lipoma of the hand extending from the thenar to the deep palmar space was reported; it was completely excised, resulting in a good surgical outcome [6]. In terms of rarity, a parosteal lipoma of the proximal phalanx of the hand accounts for less than 0.1% of all primary bone tumors. In one study, a patient with this type of lipoma was treated and managed surgically with no recurrence at the two-year follow-up [7]. The review of different articles shows that surgical excision of such lipomas is considered the preferred method that results in satisfactory outcomes; in most cases, it also restores the function of the digit. Table 1 summarizes previous studies.

**Table 1:** Attached is a table summarizing similar previous studies.

Year	Authors	Title	Aim
2013	Ramirez-Montaño L, Lopez RP, Ortiz NS.	Giant lipoma of the third finger of the hand	This case reports a rare location of lipoma of the third finger and repercussion in the decision-making process when other more common similar pathologies with varying prognoses are conceived [2].
2017	Hu Z, Yue Z, Tang Y, Zhu Y.	Lipoma of the middle finger: A case report and review of literature	The case reports a lipoma of the middle finger, and an ultrasound and MRI should be used to diagnose the finger, lipoma and excision was considered the main treatment option [4].
2017	Cemboluk Ö, Daldal İ, Topçu HN.	Giant Lipoma Of The Hand To Extending From Thenar Region To Deep Palmar Space: A Case Report	This case reports a rare giant lipoma of the hand extending from thenar to deep palmar space. Should be surgically removed due to the potential increased risk of malignancy [6].
2021	Santacoloma K, de Sá Barreto GM, Loda G, Miller MD, de Janeiro R, David R.	Giant hand lipoma: a surgical challenge	This case reports a giant hand lipoma successfully treated with a modified Bruner incision approach [1].
2021	Wafiq Wafa A, Wani S, A. Alsinan T, Alkhonizy S.	Multilocular lipoma of the left thumb of the hand: a case report	This case reports a progressive left thumb lipoma that was treated successfully with total excision and documents satisfactory outcomes [3].
2021	Sheeja RT, Bestin T, Aabha DS.	Giant Spindle Cell Lipoma of Middle Finger: Case Report and Review of Literature	This case reports an unusual location of giant spindle cell lipoma of the middle finger [5].
2021	Yadav AK, Pawar ED, Wadia F, Gs PK, Mane A, Harsoor A.	Parosteal lipoma of the proximal phalanx of hand.	This case reports a parosteal lipoma in a 45-year-old man involving the proximal phalanx of the right middle finger. The tumor was successfully excised at margins with the osseous attachment [7].

## Conclusions

While lipomas are common benign tumors, ones affecting the hand and digits are rare. This case presentation aims to contribute to the literature on lipomas, specifically articles addressing lipomas of the index and middle fingers, even if those types of lipomas are rare, to emphasize the importance of considering it as a differential diagnosis. Total excision of such lipomas is considered to be the surgical method that is preferred by most plastic and hand surgeons. This article aims to report and highlight the satisfactory outcomes after total excision of such lipomas and restoring the function as well as the cosmetic results of the hand. 
